# Bcl-xL as a Modulator of Senescence and Aging

**DOI:** 10.3390/ijms22041527

**Published:** 2021-02-03

**Authors:** Cristina Mas-Bargues, Consuelo Borrás, Jose Viña

**Affiliations:** Freshage Research Group, Department of Physiology, Faculty of Medicine, University of Valencia, Centro de Investigación Biomédica en Red Fragilidad y Envejecimiento Saludable-Instituto de Salud Carlos III (CIBERFES-ISCIII), Instituto Sanitario de Investigación INCLIVA, 46010 Valencia, Spain; cristina.mas@uv.es (C.M.-B.); jose.vina@uv.es (J.V.)

**Keywords:** aging, centenarians, senescence, Bcl-xL, senolytics, apoptosis, immunosenescence

## Abstract

Many features of aging result from the incapacity of cells to adapt to stress conditions. When cells are overwhelmed by stress, they can undergo senescence to avoid unrestricted growth of damaged cells. Recent findings have proven that cellular senescence is more than that. A specific grade of senescence promotes embryo development, tissue remodeling and wound healing. However, constant stresses and a weakening immune system can lead to senescence chronicity with aging. The accumulation of senescent cells is directly related to tissue dysfunction and age-related pathologies. Centenarians, the most aged individuals, should accumulate senescent cells and suffer from their deleterious effects, however, they enjoy a compression of morbidity. We have shown that they overexpress B-cell lymphoma-extra large (Bcl-xL). Bcl-xL could avoid an excessive burden of senescent cells through the regulation of intrinsic apoptosis, mitochondrial bioenergetics and oxidative stress. On the other hand, Bcl-xL maintains a fully functional immune system that ensures an efficient clearance of senescent cells. Moreover, there is a paradox, as inhibitors of Bcl-xL have been employed as senolytic agents, which have been shown to protect from aging in animal models. In this review, we aim to discuss how Bcl-xL could modulate senescence-associated harmful effects in centenarians, protecting them from the burden of accumulation of senescent cells.

## 1. Introduction

Humans have experienced an increase in lifespan during the twentieth century than in any other similar period in recorded history [1]. Parallel to this change, human behavior has also been modified, in part due to the social and economic development of the nowadays life style [2]. Accordingly, we are looking forward to extend the years of youth to the maximum, pushing the “old age” to the last years of our lives, where health-span extension is the principal outcome for success [3]. Therefore, we should not wait till being close to the retirement to start worrying about aging and age-related diseases, but instead, try to make the utmost benefit of aging as soon as possible and be delighted by a healthy aging.

Many features of aging result from the incapacity of cells to adapt to stress conditions. When damage accumulates irreversibly, both mitotic and postmitotic cells will undergo one of the three main pathways that will determine the pro-death or pro-survival fate of a single cell towards the benefit of the whole organism [4]. These pathways are autophagy, apoptosis and senescence. Each of them acts independently from the others, but there is more than enough evidence of a crosstalk between them. From a general point of view, the main aim is to avoid the accumulation of damage.

Initially, autophagy (self-eating) acts as a major homeostatic mechanism to eliminate damaged organelles, proteins and superfluous portions of the cytoplasm. It is known that autophagy is downregulated through aging [5] and this reduced function has been blamed for the accumulation of damaged proteins in old organisms [6,7], but it has also been reported that it contributes to cell survival and to cell death as well [8]. The fate of a damaged cell will either be apoptosis (self-killing) or senescence (irreversible growth arrest), so the damage is not propagated triggering harmful signals for the organism. What determines that a cell decides one pathway, or another is yet to be clarified.

## 2. Measuring Cellular Senescence In Vivo

Senescence is usually described as a cell fate that involves the loss of a proliferative potential, together with a resistance to cell death and an increased metabolic activity. At first, senescence is initiated by the activation of p53/p21^CIP1^ and p16^INK4a^/Rb pathways by several stresses, such as DNA damage or reactive oxygen species (ROS) among others [9]. Downstream, the spreading of these signals is mediated by ATM, IKK/NF-κB, JAK/STAT, GATA4 and mTOR signal transducers, amplifying the senescence response [10].

Once senescence is fully established in a cell, this cell shows specific morphological characteristics. A senescent cell can be identified by its enlarged size, increased lysosomal hydrolase activity (senescence associated-β-galactosidase (SA-β-gal)) and evidence of DNA damage [gamma-H2A histone family member X (γ-H2AX) foci, telomere associated-foci (TAF), senescence associated-heterochromatin foci (SAHF) and senescence associated-distension of satellite DNA (SADS)] [11]. Moreover, senescent cells display a senescence associated-secretory phenotype (SASP), which includes interleukins, chemokines, growth factors, extracellular matrix components, soluble receptors, proteases, reactive metabolites, bioactive lipids, microRNAs and extracellular vesicles [12,13].

It is very challenging to detect cellular senescence in vivo, in part due to the lack of specific biomarkers. Currently, there is not one single marker that identifies senescent cells, but a simultaneous measure of multiple parameters offers a more accurate identification of cellular senescence [14]. The most widely used markers to detect cellular senescence are listed in Table 1.

To date, the majority of in vivo studies measure senescence markers in tissue homogenates or in tissue sections. They include flow cytometry combined with histo- or cytochemical approaches, fluorescent senoprobes, and analysis of circulating SASP factors and extracellular vesicles from plasma as well [35]. However, these methods lack the ability to colocalize several biomarkers of senescence in a single cell. New approaches such as imaging flow cytometry (cytometry by time of flight, or Amnis Flow Sight) or single-cell omics would help filling this gap [36].

## 3. Senescence: From Development to Adulthood

Senescence is not a static endpoint but represents a series of progressive and phenotypically diverse cellular states acquired after the initial growth arrest. Initially, Hayflick and Moorhead introduced the term senescence to describe the phenomenon of irreversible growth arrest of human diploid cell strains after extensive serial passaging in culture [37,38]. Later, this particular type of senescence (replicative senescence) was causally linked to telomere attrition, a process that leads to chromosomal instability and promotes tumorigenesis, supporting the original hypothesis that senescence guards against unrestricted growth of damaged cells. A wealth of information about senescence in cultured cells has been acquired over the past half-century; however, senescence in living organisms is still poorly understood [39]. In part because of technical limitations for the identification and characterization of senescent cells in tissues and organs, but also due to the fact that cells in vivo are subjected to more than one senescence-inducer stressor, which complicates the comparison with in vitro studies that normally assess one single stressor at a time.

It is true that senescence is a cellular response to stress, but the fact that senescence has been detected in cells lacking metabolic alterations or DNA damage challenges the paradigm [40]. Although senescent cells might accumulate during aging and trigger age-related diseases, there is growing evidence suggesting that senescence is not the real aging of the cells. At this point, it is important to keep in mind that senescence refers to cells, while aging is at the level of the whole organism and includes physical decline, loss of memory and increased susceptibility and vulnerability to disease.

Cellular senescence is a phenomenon that has a function by itself as it displays physiological roles during embryonic development and wound healing [40]. Recent findings reveal the presence of senescent cells during embryonic development, a process called “developmental senescence” [41,42]. Interestingly, this type of senescent cells does not display any DNA damage response (DDR), preserve the ability to activate the immune system [43] and to undergo apoptosis [44]. During embryogenesis, senescent cells act as key regulators of cell proliferation and tissue remodeling, followed by immune system activation through the senescence-associated secretory phenotype (SASP) signaling in order to be quickly eliminated when their task is fulfilled [40]. Senescent natural killer (NK) cells play a role remodeling the decidua to promote the vascularization needed for fetus implantation [45]. Similarly, senescent syncytiotrophoblasts are required to maintain the viability of the placenta [46]. Taken together, the role of developmentally programmed senescence is to promote tissue remodeling and it has been proposed to be the evolutionary origin of damage-induced senescence [42].

During adulthood, senescent cells respond to different stresses and are involved in wound-healing processes. In fact, senescent cells appear at wound sites a few days after the injury, promoting optimal healing by secreting specific SASP factors [47]. Specific interleukins, such as IL-6 and IL-8, have been detected at wound sites [41]. Moreover, it has been demonstrated that the SASP factor IL-6 promotes skeletal muscle repair after injury, which in turn activates muscle stem cells to replenish the muscle [48]. In young organisms, with fully functional immune system, senescent cells are quickly cleared by macrophages and NK cells [49]. In this regard, the lifespan of senescent cells seems to be crucial; from embryogenesis to adulthood, where senescent cells are promptly removed from organs [50].

Conceptually, senescent cells can be subdivided into two main classes based on kinetics of senescence induction and functionality [51]. “Acute senescence” is induced by extrinsic stimuli that target specific cell populations, and self-organizes their clearance through SASP factors that attract the immune system effectors. Acute senescence is supposed to participate in the orchestrated biological processes to halt the expansion of specific cells and to produce specific SASP factors with concrete functions. On the opposite, “chronic senescence” appears following cellular stress or macromolecular damage. Chronic senescence is not cell-specific and is not programmed. Ineffective clearance by the immune cells and aberrant SASP factors allow the accumulation of senescent cells. The persistence of senescent cells in tissues and organs, no longer promotes tissue regeneration, but drives tissue aging and inflammation [52].

## 4. Senescence Role in Ordinary Aging

Aging is a universal, intrinsic, progressive and deleterious process [53]. Commonly, an old person’s lifespan approaches the average population lifespan; this is what we refer to as “ordinary aging”. However, there is increasing incidence of specific individuals who surpass average lifespan and approximate to the population maximum lifespan. This is the case of centenarians, exceptionally long-lived individuals who represent a model that we call “exceptional aging”.

The association between senescence and aging is supported by the increased occurrence of senescent cells obtained from old organisms [26]. Cellular senescence is now believed to contribute to organismal aging via two independent, yet not mutually exclusive, mechanisms: stem cell senescence that leads to stem cell dysfunction thus hampering tissue regenerative potential, and the SASP which causes chronic inflammation and tissue dysfunction [54]. Common cellular stresses such as oxidative stress [55,56,57], UV light [28,58] and oncogenes [59] yield senescent cells to accumulate systemically in various tissues over time contributing to tissue dysfunction in ordinary aging [60].

**Figure 1:**
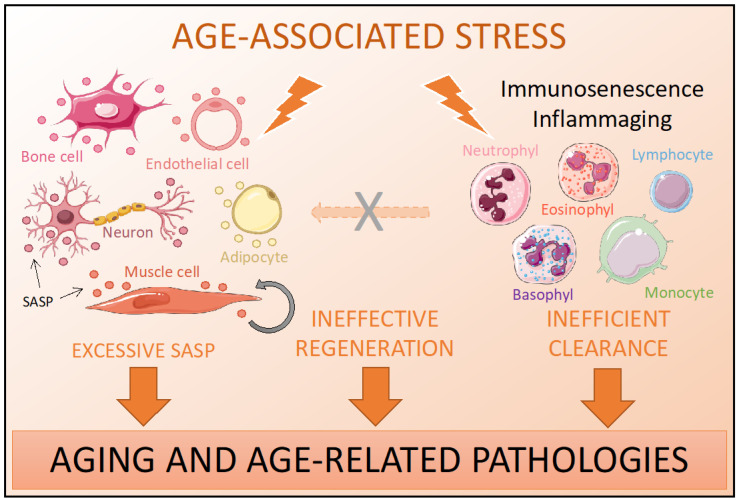
Cellular senescence in ordinary aging.

Figure 1 summarizes the impact of cellular senescence in old organisms. Senescence of immune cells hampers the clearance of non-immune senescent cells in tissues, which in turn accumulate creating a proinflammatory ambient and triggering tissue dysfunction and age-related pathologies.

### 4.1. Senescence and Age-Related Diseases

The remaining question now is, could senescent cells cause pathology? As suggested by He and Sharpless, even if senescence occurred in only 1–5% of cells within tissues, this would be a massive number of dysfunctional cells that could exert a substantial effect on host physiology [61]. Moreover, if that 1–5% of cells undergoing senescence includes a particularly important fraction of cells, then the loss of function of these cells could have a deep impact on tissue integrity and function, thus triggering the beginning of the pathology [61].

It has been described that obesity is associated with senescence [62] and contributes to type 2 diabetes [63,64]. Both aging and obesity are related to the impaired capacity of adipocyte progenitors to replicate and differentiate into fully functional, insulin-responsive adipocytes [65]. Senescent cells accumulate in adipose tissue of aged and obese humans and mice, accompanied by an increased SA-β-gal activity and an overexpression of p53, p16^INK4a^ and PAI-1, contributing to reduced adipogenesis. Moreover, senescent cells can directly cause insulin resistance through the release of SASP factors enhancing the risk of diabetes [66]. Senescence is also tightly related to frailty as it participates in osteoporosis and aggravates sarcopenia [67]. Senescent osteocytes are sixfold higher in old mice than in young mice, and the expression of SASP factors is significantly increased as well [68]. The removal of these senescent osteocytes resulted in improved trabecular bone, thicker cortices and bone strength [69]. Regarding the skeletal muscle, it is a very heterogeneous tissue, and senescence of resident cell populations, including satellite cells, fibroadipogenic progenitors, endothelial cells and immune cells contribute to muscle aging. Thus, muscle senescent cells hamper muscle regeneration and contribute to muscle loss. Similarly, cell senescence has been related to neurodegenerative disorders, such as Alzheimer’s and Parkinson’s diseases [70,71]. Emerging data have shown that neurons dissected from postmortem human brains with Alzheimer’s disease exhibit increased expression of senescent markers [72]. Importantly, experimental clearance of senescent cells led to functional improvements in mouse models of neurodegenerative diseases [73,74]. Also, senescence seems to play a role in atherosclerosis development and cardiovascular disease [75,76]. Recent studies suggest that the increased burden of senescent cells influences age-related cardiovascular dysfunction through SASP factors, which induce systemic inflammation and alter local paracrine signaling of endothelial cells [77,78]. Altogether contribute to vascular stiffness and hypertension. Finally, cellular senescence has been argued to be involved in other age-related disorders, such as hair graying, glaucoma, cachexia, osteoarthritis, cataracts and glomerulosclerosis [51,79,80,81].

### 4.2. Immunosenescence and Inflammaging

The immune system becomes deficient at old ages; while inflammaging indicates an increased pro-inflammatory activity of the innate system, immunosenescence describes the lower precision and activity of the adaptive immune system [82,83]. Immunosenescence and inflammaging are key phenomena to understand the onset of age-related diseases, which in turn accelerate the aging process and shorten the lifespan. Immunosenescence (and inflammaging) have usually a negative connotation, but, the changes of the immune system with age are complex, personalized and dynamic, being characterized by homeodynamic features balanced between “adaptive” and “maladaptive aspects” [84].

Senescent cells produce very large quantities of many bioactive compounds as part of their SASP. These secreted factors have paracrine effects on surrounding cells. The SASP can induce accelerated aging by establishing a chronic inflammatory microenvironment [85]. In mouse models, the SASP has been associated with several age-related diseases such as atherosclerosis [76], sarcopenia [86], Alzheimer’s disease [70], cataracts [87] and intervertebral disc degeneration [88]. Removal of senescent cells in these tissues reduces local inflammation and rescues some of the associated tissue impairments [76,89]. The SASP is also intended to create a proinflammatory milieu that facilitates the removal of senescent cells [90,91]. However, immune cells undergo senescence during aging, thus hampering this mechanism and allowing senescent cells to become permanent along with their adverse effects [92]. Additionally, senescent cells in old organisms seem to be able to escape immune elimination by T cells [93]. Thus, the increased incidence of senescent cells in tissues at old ages, is the result of accumulated cellular damage together with a dysfunctional immune system that drives an inefficient clearance. The accumulation of senescent cells has been thought to trigger aging, but it could also suggest that senescent cells accumulate as a result of the aging process itself [94].

The combination of an inefficient clearance, excessive SASP and ineffective regeneration may explain the chronicity of senescent cells during aging, thus contributing to the aging phenotype with increased morbidity and mortality in the elderly [95,96].

## 5. Senescence Role in Exceptional Aging

Centenarians are a model of exceptional aging because they live longer than their counterparts, and more importantly, because they compress morbidity, living up to 100 disease-free years. From a general point of view, cellular senescence physiological main function is to promote tissue remodeling through a stable proliferative arrest, a secretory phenotype that recruits immune cells and modifies the extracellular matrix, and the mobilization of nearby progenitors that repopulate the tissue. This sequence of events is referred to as senescence-clearance-regeneration [75]. Moreover, a recent study reported a significant relationship between longevity and the capacity to induce senescence following DNA damage in primary fibroblast cultures of six long-lived mammalian species; thus, suggesting that senescence has a pro longevity role [97]. This finding supports the idea that a specific grade of senescence may have positive effects for the organism. According to this, centenarians seem to be able to avoid a chronic accumulation of senescent cells, and benefit from an acute senescence [98].

### 5.1. Balance between Senescence and Apoptosis in Exceptional Aging

Senescence and apoptosis are alternative cell fates that can be triggered by the same stimuli. Although it is still not clear what makes the cell decide between one pathway or the other, mechanisms must be in place to lock those decisions. Previously, we performed a transcriptomic analysis of centenarians, septuagenarians and young people’s blood. Sub-network classification of the differentially mRNAs levels revealed that all the signaling pathways involved in successful aging were associated to three apoptosis-related genes: *Bcl-xL*, *Fas* and *Fas ligand* (*FasL*) [99].

Bcl-xL is an anti-apoptotic protein that inhibits the intrinsic (mitochondrial) pathway to apoptosis. Bcl-xL promotes cell survival by migrating to the mitochondrial outer membrane, counteracting mitochondrial permeabilization and the subsequent cytochrome c release. The latter event is critical for the formation of the Apaf-1/Caspase-9 apoptosome, which unleashes a cascade of caspase activations that culminate in apoptosis. Fas and FasL are mainly involved in the control of the extrinsic (receptor-mediated) pathway to apoptosis. The binding of Fas ligand to Fas receptor results in the binding of the adapter protein FADD which in turn associates with procaspase 8. At this point, a death-inducing signaling complex (DISC) is formed, resulting in the auto-catalytic activation of caspase 8.

During senescence, both apoptotic pathways could be differentially modulated with variable impacts on the aging process. As previously suggested by Franceschi et al., a well-balanced modulation of apoptosis may be useful to expand lifespan, and to reduce age-related degenerative and inflammatory diseases [100]. The fact that centenarians overexpress *Bcl-xL*, *Fas* and *FasL*, suggests a very fine-tuned control of apoptosis. Cells with damage might be more prone to enter senescence and become apoptosis resistant by overexpressing *Bcl-xL*. On the other hand, Fas and FasL may drive cells accumulating environmental damage to enter apoptosis avoiding senescence [33]. This would prevent an excessive burden of senescent cells. Supporting this hypothesis, lymphocytes from healthy centenarians have shown reduced expression levels of senescence biomarkers when compared to old donors [98].

### 5.2. Bcl-xL Modulates Senescence in Exceptional Aging

Despite its well-documented anti-apoptotic role, Bcl-xL is also related to mitochondrial bioenergetics by modulating mitochondrial fusion and fission, increasing total mitochondrial biomass and enhancing the efficiency of the ATP synthesis by decreasing the proton leak within the F1F0 ATPase [101,102]. As cellular senescence can be both beneficial and detrimental for the organism, accordingly, Bcl-xL might play a dual role on senescence.

A possible hypothesis could be that during acute senescence, Bcl-xL effects on mitochondria would help senescent cells to cover their metabolic demand to secrete the SASP to promote their clearance as part of the senescence-clearance-regeneration procedure. However, senescent cells are also characterized by dysfunctional mitochondria, due to an imbalance between mitochondrial fission and fusion, which is critical for the functionality of the mitochondrial network [103,104]. In this scenario, Bcl-xL might avoid the accumulation of dysfunctional mitochondria in senescent cells, thus preventing their detrimental effect on tissue homeostasis.

Regarding the first part of the hypothesis, senescent cells are known to display metabolic changes such as increases in glycolysis and mitochondrial metabolism [105]. The increased SASP production and secretion relies on enhanced ATP production mediated by mitochondrial metabolism and glycolysis. Bcl-xL increases the efficiency of ATP synthesis by decreasing the proton leak within the F1F0 ATPase, thus improving mitochondrial metabolism [106]. Furthermore, it has been demonstrated that Bcl-xL overexpression protects against oxidative stress-induced apoptosis [107]. Supporting the second part of the hypothesis, a recent study revealed the existence of mitomiRs (nuclear-coded miRNAs found within mitochondria [108]), such as miR-181-a, miR-34a and miR146a, that can target Bcl-xL to modulate the mitochondrial fusion and fission dynamics in senescent cells [109]. This process has been implicated in the quarantining of impaired mitochondria during mitophagy [110,111,112].

The role of autophagy in senescence is still controversial. Some authors suggest that senescent cells engage autophagy with protein synthesis to support the amino acid demand [113]. Other authors support that defective mitophagy leads to accumulation of dysfunctional mitochondria and ROS-induced senescence [114]. Several findings have shown that Bcl-xL can influence macroautophagy through binding to the autophagy regulator Beclin-1 and blocking its participation in the triggering of autophagosome formation [115,116]. Similarly, Bcl-xL antagonizes Parkin-dependent mitophagy in a Beclin-1-independent manner [117]. Taken together with its role as a regulator of mitochondrial fusion/fission dynamics, enhancer of the F1F0 ATPase and protector of ROS overproduction, Bcl-xL acts as a global regulator of mitochondrial homeostasis.

Consistent with this, Bcl-xL seems to play a key role in the regulation of senescence, either directly or indirectly, through the modulation of intrinsic apoptosis, mitochondrial bioenergetics, oxidative stress and autophagy [101]. Since Bcl-xL could be a modulator of the harmful consequences of senescent cells accumulation, it would be of utmost interest to assess whether Bcl-xL might also modulate SASP factors or senescence-derived extracellular vesicles cargo.

### 5.3. Bcl-xL Maintains Immunosurveillance of Senescent Cells in Exceptional Aging

Clearance efficiency matters in order to avoid senescent cells accumulation and to prevent the risk for age-related diseases [61]. To this end, senescent cells modulate NK cell effector functions through the SASP and extracellular vesicles [52]. But, as we age, the immune system also weakens by two main causes: hematopoietic stem cell (HSC) dysfunction, and cellular senescence of immune cells such as macrophages [118]. HSC dysfunction leads to reduced NK cell activity [119], which in turn contributes to the accumulation of senescent cells. Remarkably, healthy elderly individuals as well as centenarians show an increase of total NK cells [120]. Macrophages are also in charge of the clearing of senescent cells. Reduced chemotaxis might be involved in impaired capacity of macrophages to respond to SASP factors and to migrate to the places where senescent cells accumulate [50].

Long-lived organisms are equipped with an efficient immune system that eliminates senescent cells [121]. Such a process would enable them to avoid or delay age-related diseases and to live longer. As stated before, centenarians overexpress Bcl-xL, which is important for the development and maintenance of the immune system [122]. Our previous analysis revealed that leukocyte chemotaxis and NK cell activity was significantly impaired in septuagenarians in comparison with young people and centenarians [99]. Moreover, transduced lymphocytes from septuagenarians with Bcl-xL expressed lower levels of senescence markers and increased their proliferation capacity compared to controls. These results suggest that overexpression of Bcl-xL is able to rescue impaired lymphocytes from septuagenarians to behave like those from centenarians and young individuals. Accordingly, there is a positive correlation between the amount of total NK cells and the health status of elderly individuals [120]. Furthermore, it has been demonstrated that Bcl-xL is related to the development of B cells and is highly expressed in T cells, which is necessary for their function [123]. Consistent with this, Bcl-xL plays an important role in maintaining the effectiveness of the immune system during aging.

**Figure 2:**
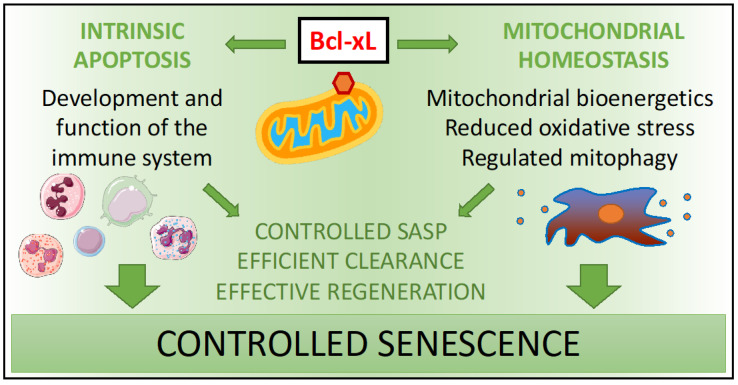
Bcl-xL modulation of senescence.

Taken together, Bcl-xL overexpression in centenarians modulates cellular senescence according to the homeostatic needs of the organism. On the one hand, Bcl-xL improves the function of the immune system for an effective clearance of senescent cells. On the other hand, Bcl-xL controls the SASP production by maintaining mitochondrial homeostasis and regulating autophagy (see Figure 2).

## 6. Bcl-xL as a Senolytic

Senotherapeutics are a new class of drugs that selectively kill senescent cells (senolytics) or suppress their SASP (senomorphics or senostatics) without killing the senescent cell [10]. Senescent cells mainly depend on the immune system to be cleared; thus, a dysfunctional immune system will lead to accumulation of senescent cells within tissues. To promote the depletion of senescent cells, senolytic drugs aim to eliminate senescent cells without affecting quiescent or proliferating cells [124]. The clearance of senescent cells has been shown to delay and reduce the aging phenotype in several tissues of premature and natural aged models, such as BubR1 and Ercc1 knockout progeroid mice [125].

Since the expression of anti-apoptotic and pro-apoptotic genes is higher in senescent cells compared to healthy cells, inhibitors of Bcl-xL have been described as senolytic agents because they only induce apoptosis in senescent cells, both in vitro and in vivo [126]. ABT737, ABT263 or Navitoclax, which targets the Bcl-2/Bcl-xL proteins, is a potent senolytic drug that selectively kills senescent cells, regardless of how they were induced [127,128]. Initially, Navitoclax was introduced as a broad spectrum senolytic because it killed more than 60% of senescent human umbilical vein endothelial cells (HUVECs) and senescent WI-38 and IMR90 human lung fibroblasts [127], as well as MEFs in 72h [128]. However, Navitoclax had no apoptotic effect on senescent pre-adipocytes [62]. Moreover, a phase II study revealed that the administration of Navitoclax has some side effects, thrombocytopenia and neutropenia among them [129]. These well-known side effects emphasize the importance of Bcl-xL in the survival of platelets and neutrophils in a normal setting [130].

Although senolytics show a wide range of beneficial effects for senescence-related indications, especially on cardiovascular function, osteoporosis and frailty [96], the use of senolytics as an anti-aging treatment still has some limitations. First of all, senolytic therapies are still not effective in all senescent cell types and should be tissue specific. Next, there is no information regarding the replacement of senescent cells by new cells. Excessive clearance of senescent cells might speed up stem cell exhaustion. If there are no cells to fill the empty place, in this case, it might be better to keep the senescent cell rather than no cell [40].

Taken together, more research is needed to evaluate in vivo senescence to determine the beneficial or harmful consequences of senescent cell elimination by senolytic agents. Centenarians are the proof of concept that senescence can be also beneficial throughout our lives.

## 7. Conclusions and Future Perspectives

Senescence is a physiological response with a particular role from embryonic development until adulthood. The organism benefits from a brief senescence, where senescent cells are efficiently cleared to enable tissue regeneration. Chronic senescence appears upon aging as a result of the accumulation of senescent cells as a consequence of constant damage through life and an impaired clearance by the immune system.

Centenarians overexpress Bcl-xL [99], which plays a role in the regulation of senescence, either directly or indirectly, through the modulation of intrinsic apoptosis, mitochondrial bioenergetics, oxidative stress and autophagy [101]. In this scenario, centenarians have a fine-tuned control of apoptosis which enables them to avoid an excessive burden of senescent cells, together with a fully functional immune system that removes senescent cells on time. Thus, centenarians avoid tissue functional deterioration and prevent age-related pathologies due to a good control of senescent cell accumulation. In other words, centenarians’ ability to modulate senescence might explain, at least in part, their morbidity compression. Moreover, centenarians’ intrinsic capacity might be related to their high regenerative potential to replace senescent cells by new cells. Altogether, it looks like that centenarians still benefit from an acute senescence.

The principles of regenerative medicine can be applied to aging and age-related diseases. Expression of the pluripotency factors (*SOX2*, *OCT4*, *KLF4* and *c-MYC*) in senescent cells has been shown to allow cell cycle entry with reset of telomere size and gene expression profiles, to reduce oxidative stress and to switch mitochondrial metabolism [131,132]. Bcl-xL enhances Yamanaka factors-induced iPSC generation from adult peripheral blood mononuclear cells (PBMCs) [133], future research is needed to study the possible role of Bcl-xL cellular reprogramming.

These newly recognized beneficial signaling functions of senescence suggest that indiscriminately targeting senescent cells or modulating their secretome for anti-aging therapy may have negative consequences [81]. Similarly, senescent cells clearance may also have adverse outcomes, especially in young individuals [134]. Both pro-senescent and anti-senescent approaches can be desirable depending on the therapeutic context. Pro-senescent therapies can be useful for cancer treatment and for ongoing tissue repair processes such as organ fibrosis, whereas anti-senescent therapies can be beneficial to eliminate the tissue-specific burden of senescent cells that accumulate during aging or chronic damage in order to prevent age-related pathologies [75]. In this regard, studying the role of Bcl-xL on the modulation of senescence might have an impact in the field of aging, as this protein is overexpressed in the individuals who live longer and better, i.e., centenarian ones.

## Figures and Tables

**Table 1:** Markers of cellular senescence.

Marker	Senescence	Ref.
Proliferation (Ki67, BrdU assay)	Absent	[15,16]
Cell cycle inhibitors (p21^CIP1^, p16^INK4a^)	Increased	[17,18,19]
DNA damage (p53, γH2AX foci, TAF, SAHF, SADF)	Increased	[20,21,22,23,24,25]
SA-β-gal activity	Increased	[26,27,28]
Lamin B1	Decreased	[29]
Nuclear exclusion of HMGB1	Present	[30]
SASP factors (IL-1, IL-6, IL-8, PAI-1, MMPs)	Present	[31,32]
Anti-apoptotic proteins (Bcl-xL, MCL-1)	Increased	[33,34]

## Data Availability

Not applicable.

## Abbreviations

γH2AX	Gamma H2A histone family member X
ATM	Ataxia Telangiectasia Mutated
Bcl-xL	B-cell lymphoma extra-large
DDR	DNA damage response
DISC	Death-inducing signaling complex
FADD	Fas-associated protein with death domain
GATA4	GATA binding protein 4
HMGB1	High mobility group box 1
HSC	Hematopoietic stem cells
HUVEC	Human umbilical vein endothelial cells
IFNG	Interferon- γ
IKK/NF-κB	IK kinase/Nuclear factor κB
IL	Interleukins
iPSC	Induced pluripotent stem cell
JAK/STAT	Janus kinase/Signal transducer and activator of transcription
MEFs	Mouse embryonic fibroblasts
MMPs	Matrix metalloproteinases
NK	Natural Killer
PAI-1	Plasminogen activator inhibitor 1
PBMC	Peripheral blood mononuclear cells
PI3K	Phospho-Inositol 3 Kinase
ROS	Reactive oxygen species
SA-β-gal	Senescence associated- β-galactosidase
SADS	Senescence associated- distension of satellite DNA
SAHF	Senescence associated- heterochromatin foci
SASP	Senescence-associated secretory phenotype
SP1	SP1 transcription factor
TAF	Telomere associated- foci
TCR	T-cell receptor
TGF β1	Transforming growth factor- β1
TNF	Tumor necrosis factor
